# Effects of Parkinson’s disease on mechanical and microstructural properties of the brain

**DOI:** 10.1016/j.nicl.2025.103857

**Published:** 2025-08-05

**Authors:** Christoffer Olsson, Mikael Skorpil, Per Svenningsson, Rodrigo Moreno

**Affiliations:** a Department of Biomedical Engineering and Health Systems, KTH Royal Institute of Technology, Stockholm, Sweden; b Department of Molecular Medicine and Surgery, Karolinska Institutet, Stockholm, Sweden; c Department of Neuroradiology, Karolinska University Hospital, Stockholm, Sweden; d Department of Clinical Neuroscience, Karolinska Institutet, Stockholm, Sweden; e Department of Neurology, Karolinska University Hospital, Stockholm, Sweden; f Department of Neurobiology, Care Sciences and Society, Karolinska Institutet, Stockholm, Sweden

**Keywords:** Parkinson’s disease, Magnetic resonance elastography, Multidimensional diffusion MRI, Microstructure, Diffusion MR

## Abstract

Magnetic Resonance Elastography (MRE) is a novel technique to study the brain by measuring its mechanical properties, such as stiffness and viscosity. These properties may provide insights into how the microstructure of the brain changes due to a pathology, however the connection between these microstructural mechanisms and the measured biomechanical properties are still largely unknown. For this reason, the present exploratory study utilizes multidimensional diffusion magnetic resonance imaging (MD-dMRI), apart from MRE, to extract microstructural parameters of the whole brain tissue for a small cohort of 12 Parkinson disease (PD) patients and 17 healthy controls. A combination of these methods provides valuable insights into subtle changes due to PD as it probes variables such as microscopic fractional anisotropy (μFA) combined with measures of shear stiffness. MRE and MD-dMRI quantities across the brain are compared between the two groups and analyzed. It was found that there were significant softening effects in the temporal and occipital lobes due to PD, associated with an increase in the mean diffusivity in those regions, whereas other microstructural properties remained largely unchanged. The mesencephalon, on the other hand, displays changes in the MD-dMRI parameters consistent with neuronal atrophy, however no softening of this region was detected. In most regions, stiffness is significantly reduced due to age, which is correlated with a decrease in μFA and increase in MD. We hypothesize that age effects can mostly explain neuronal atrophy, whereas softening due to PD effects involve additional mechanisms.

## Highlights

• The occipital and temporal lobes decrease most in stiffness due to PD.
• The cerebrum softens due to aging effects coupled to neuronal atrophy.
• The mesencephalon does not soften due to PD but show signs of neuronal atrophy.
• The stiffness across the whole brain are most strongly correlated with MD and µFA.

## Graphical abstract

**Figure:**
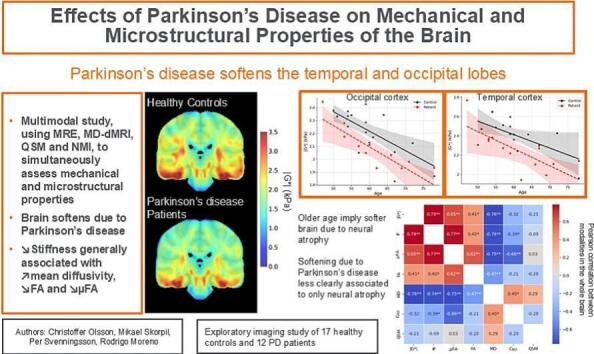

## 1 Introduction

Parkinson’s disease (PD) is a neurodegenerative disease that affects about 1 % of the population over the age of 60 (Bloem et al., 2021, Tysnes and Storstein, 2017). It is characterized by a progressive loss of dopaminergic neurons in the substantia nigra, which causes motor function impairments, such as bradykinesia, rigidity and tremor. Apart from motor functions, PD patients typically also exhibit other non-motor symptoms, such as cognitive decline and depression. Traditional MRI techniques have revealed substantial macrostructural changes in various brain regions due to atrophy in PD (Pieperhoff et al., 2022), especially in the advanced stages of the disease. Less conventional techniques, such as quantitative susceptibility mapping (QSM) and neuromelanin imaging (NMI), have also been used to depict differences between PD and healthy controls (Andersson Forsman et al., 2024, Biondetti et al., 2021, He et al., 2023, Sjöström et al., 2017) by quantifying the amount of iron and neuromelanin, respectively, in various areas of the brain. Any alterations due to PD in macroscopic measures arise as a consequence of microstructural changes in the brain. Thus, assessing microstructural changes in PD might give hints of the mechanisms of the disease that lead to macrostructural changes, such as volumetric reduction of brain tissue, iron, or neuromelanin content.

A method of obtaining microstructural information is multidimensional diffusion magnetic resonance imaging (MD-dMRI), which is an advanced diffusion sequence that combines both a multi-shell diffusion MRI (dMRI) acquisition and a spherical b-tensor encoding to obtain different microstructural parameters, such as microscopic anisotropy or free water fractions and has previously been used to investigate PD (Kamiya et al., 2020, Surova et al., 2016, 2018). The main advantage of this MRI modality is that it can distinguish between different structural configurations at the microscale that are impossible to detect with standard processing of dMRI data.

Furthermore, the mechanical properties (e.g. stiffness and viscosity) of the brain can provide additional insights into the brain’s microstructure. Thus, investigations on how these properties change due to PD may provide valuable information about the disease. For example, studies on Alzheimer’s disease, have shown that changes in the mechanical properties, as measured by magnetic resonance elastography (MRE) (Muthupillai et al., 1995), can appear before they are visible in structural MRI (Hiscox et al., 2020). MRE is a technique that can estimate the mechanical properties of the brain in vivo, in particular, shear stiffness and viscosity. For MRE, the subject’s head is gently vibrated inside an MR scanner, while a set of motion encoding gradients are employed to detect small displacements of the tissue from the phase of the MR signal. A map of the mechanical properties of the brain can then be reconstructed based on these displacement images. MRE has been used to study several different aspects of the brain and is emerging as an increasingly promising imaging modality (Arani et al., 2021, Feng et al., 2024). For example, studies have found that the brain generally softens with increasing age (Hiscox et al., 2021, Sack et al., 2009). Moreover, it has been found that the mechanical properties of the brain are affected by pathologies like Alzheimer’s disease (Hiscox et al., 2020, Murphy et al., 2011), multiple sclerosis (Herthum et al., 2022, Wuerfel et al., 2010), and PD (Lipp et al., 2013, 2018).

The mechanical properties of the brain measured by MRE at a voxel-based level are determined by the microstructural properties of the brain, i.e., by its structural composition of neurons, glial cells, blood vessels, and extracellular water, among others. Extensive efforts have been made to study how the microstructure relates to the stiffness of healthy and diseased brain tissue (Braun et al., 2024, Hain et al., 2016, Lu et al., 2006, Schregel et al., 2012). Still, the understanding of microstructure and mechanics of the brain *in vivo* is limited due to few studies utilizing imaging techniques that extract such properties.

This study contributes to closing the gap between macro and microstructural information by assessing the relationships between the mechanical properties estimated with MRE and microstructural parameters estimated with multidimensional diffusion MRI (MD-dMRI) in PD. Apart from these, we also used quantitative susceptibility mapping (QSM) and neuromelanin imaging (NMI). To the best of our knowledge, this is the first study where MD-dMRI has been employed to provide a microstructural explanation for macroscopical property changes measured with MRE, QSM, and NMI on PD.

## 2 Methods

### 2.1 Subject information

A description of the population is shown in Table 1. The cohort consists of 17 healthy controls (HC) and 12 PD subjects in ON state. The disease severity of the PD subjects was evaluated by a clinician using the Unified Parkinson’s Disease Rating Scale (MDS-UPDRS I-IV). We obtained a written informed consent form prior to the participation from all subjects and obtained ethical approval from the Swedish Ethical Review Authority (Dnr. 2022-03209-02).

**Table 1:** Subject information.

**Subject Info**	**Controls**	**PD patients**
Mean age ± SD (min–max)	60 ± 6 (50–78)	63 ± 9 (47–78)
Gender (F:M)	3:14	3:9
Disease Duration (Years)	−	6.4 ± 5.3 (1–17)
LEDD (mg)	−	479 ± 301 (72–958)
H & Y	−	1.75 ± 0.45 (1–2)
MoCA	−	28.9 ± 1.2 (27–30)
MDS-UPDRS-1	−	7.0 ± 2.3 (2–10)
MDS-UPDRS-2	−	6.5 ± 2.3 (3–11)
MDS-UPDRS-3	−	18.7 ± 11.0 (4–41)
MDS-UPDRS-4	−	5.1 ± 4.1 (0–12)
Total MDS-UPDRS	−	35.3 ± 9.8 (24–58)

Values listed as means ± SD (min–max). Clinical ratings for PD patients includes disease duration in years, Levodopa equicalent daily dose (LEDD) in mg, Hoehn and Yahr scores (H & Y), Montreal Cognitive Assesment (MoCA) scores, and MDS-UPDRS scores.

### 2.2 Image acquisition

All subjects were scanned using a Philips Ingenia CX 3T scanner with a 32-channel head coil. Participants’ heads were placed on top of a pneumatically driven vibrating pillow that was used for the MRE scan at the end of the session. The session included standard T1-weighted and T2-weighted images. T1w: using a gradient echo sequence with a preparation pulse for contrast enhancement (Turbo Field Echo), TR/TE: 6.7/3 ms, 1 × 1 × 1 mm^3^ resolution, full head FOV, T2w: TR/TE: 3000/280 ms, 1 × 1 × 1 mm^3^ full head FOV. MD-dMRI images were acquired as explained in (Topgaard, 2017) and (Szczepankiewicz et al., 2019) with TR/TE: 4000/111 ms and a resolution of 2.5 × 2.5 × 2.5 mm^3^, using spherical and linear b-tensors with 5 b-values (b = 0, 100, 700, 1400, 2000 s/mm^2^). The MD-dMRI acquisition lasted for a total of 6.5 min. MRE images were acquired using a spin-echo EPI sequence with 3-directional motion encoding gradients, with a gradient strength of 70mT/m, TR/TE: 4800/67 ms, 3 × 3 × 3 mm^3^ with full brain FOV.^1^ The driver pillow was set to vibrate with a driving frequency of 60 Hz and was provided by Mayo Clinic (see, e.g., (Kruse et al., 2008) for more details on the acquisition protocol). The acquisition lasted for about 4.5 min.

To obtain QSM images, 8 different gradient-recalled-echo (GRE) images with different echo times linearly spaced between 9.8 ms-57.4 ms were acquired with a TR of 62 ms, a flip angle of 15° and a spatial resolution of 1x1x1 mm^3^.

NMI was obtained with a gradient echo sequence with a FOV of 210 × 210 × 46.8 mm^3^ with 36 1.3 mm thick slices centered at the midbrain and in-plane resolution of 0.6 mm, TR/TE: 83/7.7 ms and a flip angle of 16°. The acquisition lasted for about 5.25 min. The obtained NMI-intensity was finally normalized to NM-CNR = $\frac{\text{< N}{\text{M}}_{\text{ROI}}\text{> -< N}{\text{M}}_{\text{REF}}\text{>}\;}{\text{< N}{\text{M}}_{\text{REF}}\text{>}\;}$ where < NM_ROI_ > is the mean intensity within the region of interest, and < NM_REF_ > is the mean intensity within a reference region typically containing low levels of neuromelanin and exhibit low inter-subject variability (Ben Bashat et al., 2022). In this study, the WM-temporal was chosen as reference as this is a large white-matter region fully contained within the FOV of the NMI.

Each scanning session lasted about 45 min and included other imaging modalities not relevant to the work of this paper, all acquired before the MRE scan.

### 2.3 Postprocessing

#### 2.3.1 MRE

The acquired MRE displacement images were inverted to viscoelastic parameter maps as described in (Oliphant et al., 2001). This results in an image with the complex shear modulus per voxel, which is defined as G*=G′+iG″. Stiffness is defined as |G*|, and the viscosity-related phase angle φ = arctan(G′′/G′).

The acquired displacement images from the MRE scans were inverted to viscoelastic parameter maps using a direct inversion algorithm based on the Helmholtz equation, as originally described in (Oliphant et al., 2001). This method estimates the complex shear modulus G* = G′ + iG″, from which the magnitude (|G*|), representing stiffness and the phase angle φ = arctan(G′′/G′), representing relative viscosity are computed. This specific direct inversion method calculates the curl of the displacement field, to remove the longitudinal component of the wave motion, before solving the Helmholtz equation under assumptions of isotropy and local homogeneity. A more detailed description of this method can be found in more detail in the following references (Murphy et al., 2012, 2013).

#### 2.3.2 MD-dMRI

**Fig. 1:**
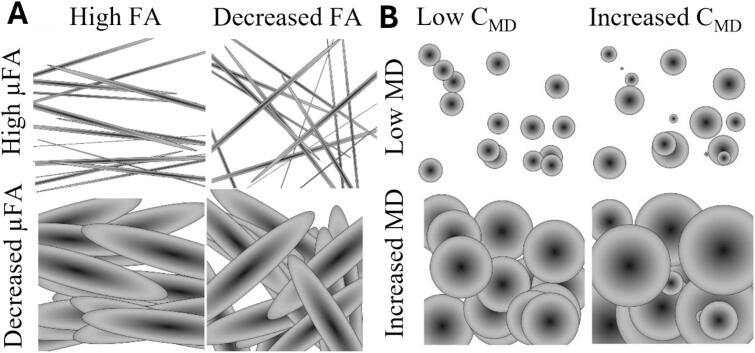
Schematic for how subvoxel properties change with varying MD-dMRI parameters: A) FA vs.µFA and B) MD vs. C_MD_ for different sub-voxel scenarios. A) high µFA means that there are plenty of water compartments that diffuses with high anisotropy, and by altering the alignment of these compartments one can obtain either high or low average anisotropy (FA). Decreasing µFA implies either that there are fewer compartments of high anisotropy, or that the anisotropy of the compartments decreases. B) MD measures the mean diffusivity of the entire voxel and the C_MD_ is a measure for the normalized variance of MD for the different subpopulations within the voxel.

Regarding MD-dMRI, Synb0-DISCO (Schilling et al., 2019, Schilling et al., 2020, 2020) was used to produce synthetic distortion correction images for the MD-dMRI images for subsequent topup correction using FSL (Andersson et al., 2003, Andersson and Sotiropoulos, 2016). The images were post-processed using the open-source MD-dMRI software (Nilsson et al., 2018) to estimate four parameters: fractional diffusivity (FA), mean diffusivity (MD), microscopic FA (μFA) and the normalized variance of MD (C_MD_). See (Westin et al., 2016) for more details. From these, μFA and C_MD_ cannot be estimated with standard diffusion MRI (dMRI). These parameters were estimated using methods described in (Westin et al., 2016). Fig. 1a sketches the difference between FA and μFA. FA is conventionally obtained from standard dMRI; however, this parameter is a macroscopic average measure of anisotropy; thus, if a volume contains, e.g., axons with different orientations, the average anisotropy will be very low. In contrast, μFA measures microscopic anisotropy; thus, the same volume would result in a high μFA. If the volume has a high density of aligned fibers, both FA and μFA are high. In turn, if the voxel exhibits high MD (see Fig. 1b), this means that the volume contains a lot of freely diffusing water molecules, and if there is a low C_MD,_ the obtained MD signal is drawn from a homogeneous distribution of water molecules with similar diffusivity, however, if the C_MD_ is high this distribution is inhomogeneous; the latter means that there are several different populations of water molecules within the volume (e.g., intra- and extracellular water) that diffuses with different rates.

#### 2.3.3 QSM

The images acquired with the QSM protocol were postprocessed with the use of the morphology-enabled dipole inversion (MEDI)-toolbox (Liu et al., 2011) to invert the phase images and obtain the final QSM.

#### 2.3.4 Spatial normalization

All parameters extracted from MRE, MD-dMRI, QSM and NMI were, after postprocessing, registered to subject-specific T1 images (1 × 1 × 1 mm^3^), which had been parcellated into the Desikan-Killiany atlas (Desikan et al., 2006) with the use of FreeSurfer v7.2. Values from substructures in the brainstem (mesencephalon and pons) were also extracted using an additional segmentation method within FreeSurfer (Iglesias et al., 2015). These regions were then combined into various groups (e.g., the cortical layer of the occipital lobe). Median values of each of these regions were then extracted for statistical analysis.

To analyze subcortical structures we utilized a high-resolution atlas (in MNI space) of the following structures (Pauli et al., 2018):

• Putamen (Pu)
• Caudate Nucleus (Ca)
• Nucleus Accumbens (NAC)
• Globus Pallidus external and internal (GPe and GPi)
• Substantia Nigra pars compacta and pars reticulata (SNc and SNr)
• Red Nucleus (RN)
• Ventral Tegmental Area (VTA)

The subject-specific T1w images were first registered to an MNI template using affine registration, followed by a nonlinear registration with the use of ANTs (Avants et al., 2008). The inverses of these registrations were then applied to the subcortical atlas to obtain the subject-specific parcellation of the subcortical structures in the subject space.

### 2.4 Statistical analysis

**Fig. 2:**
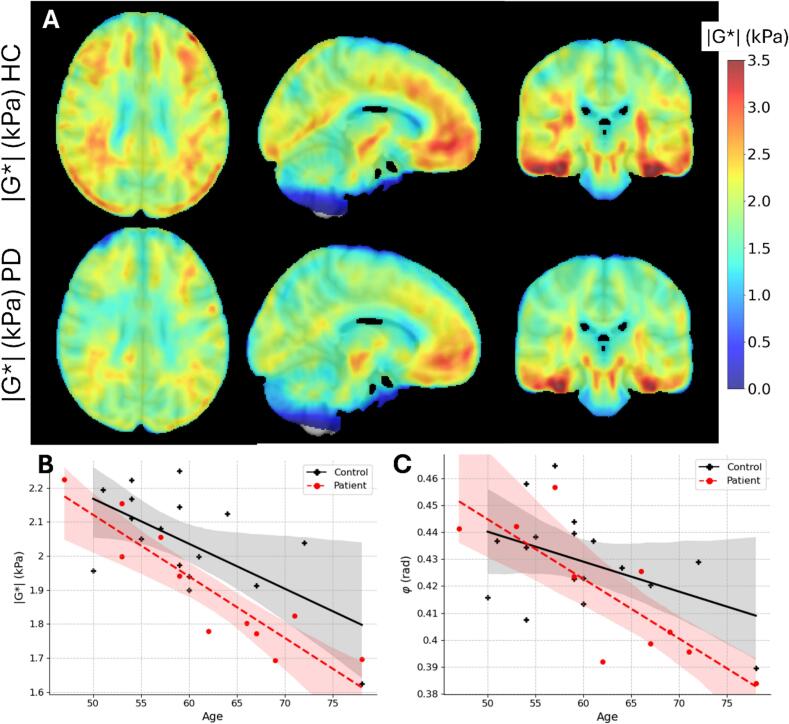
A) Brain stiffness averaged for all subjects in MNI space. Top: healthy controls. Bottom: PD patients. Columns show axial, sagittal, and coronal views, respectively. The scale bar shows stiffness values given in Pa. B) Average whole brain stiffness |G*| and C) viscosity angle φ (in radians) vs subject age for healthy controls (black points) and patients with PD (red points). Solid lines show a linear fit to either the PD (red dashed line) or HC (black solid line) and shaded areas show the 95% confidence interval. Regions containing CSF were excluded from the average.

**Fig. 3:**
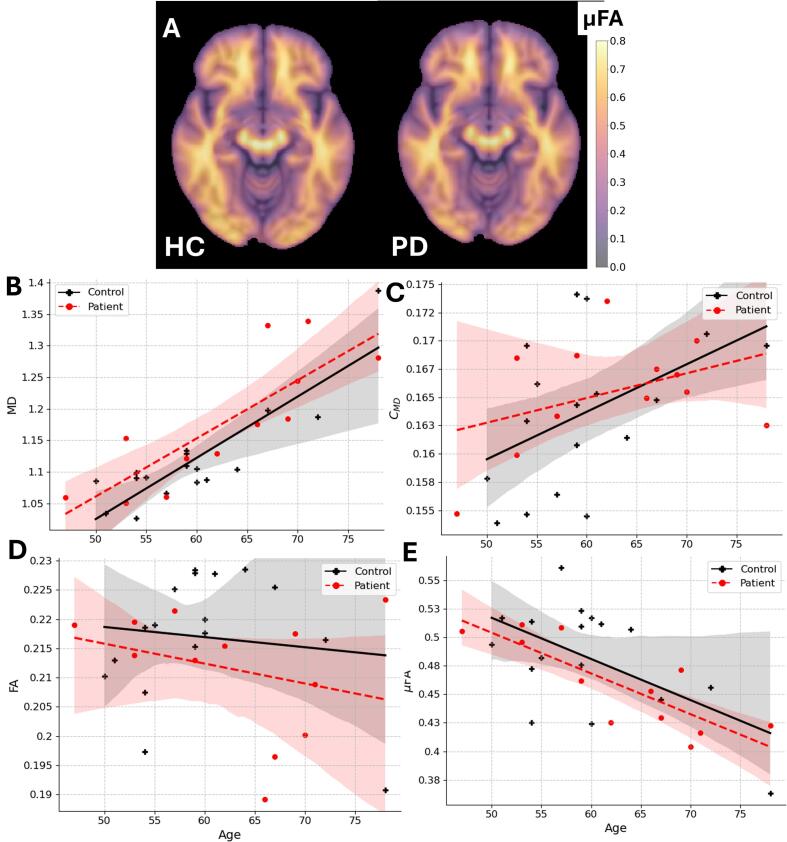
A) MD-dMRI parameter example maps for μFA averaged over all healthy controls and all PD patients registered to the MNI space. B) – E) -MD-dMRI parameters averaged over the whole brain as a function of age. Regions containing CSF are excluded. Black and red points indicate healthy controls and PD patients, respectively. Linear fits are shown as a general trend line for the different groups and shaded areas show the 95% CI.

**Fig. 4:**
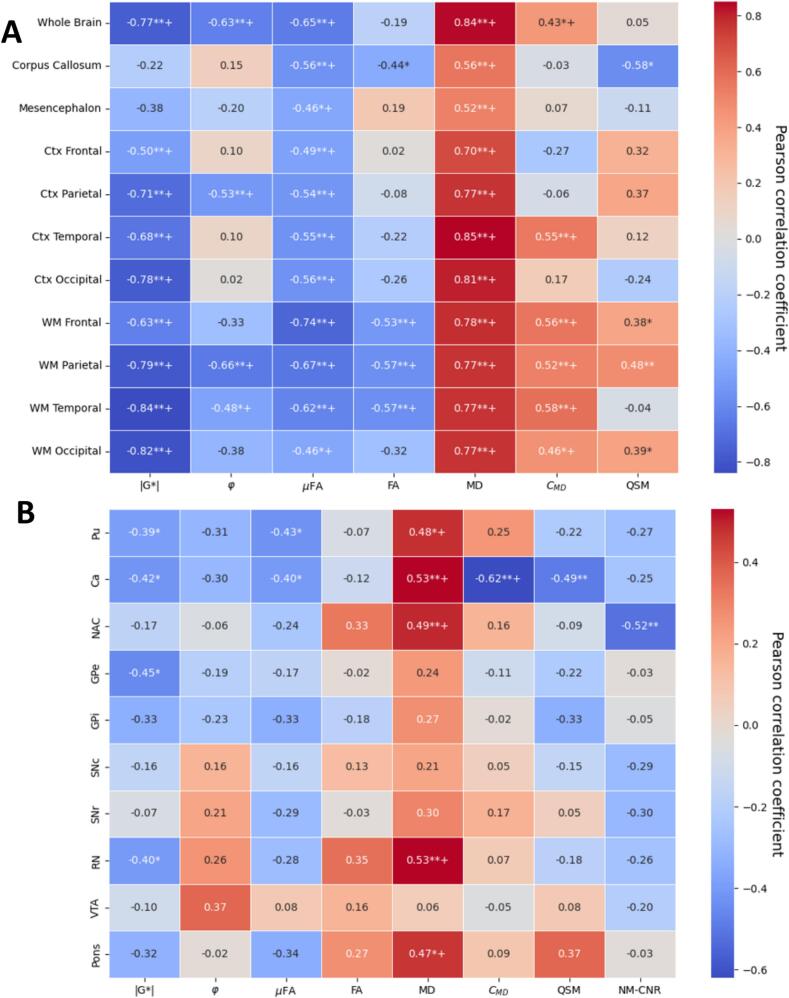
Age effects. Pearson correlations between age and median intensity of A) major brain regions and B) subcortical regions, corrected for group effects (PD or HC). (*=p < 0.05. **=p < 0.01. += p_FDR-corr < 0.05). Pu=Putamen, Ca=Caudate Nucleus, NAC=Nucleus Accumbens, GPe/i = Globus Pallidus external/internal, SNc/r=Substantia Nigra pars compacta/reticulata, RN = Red Nucleus, VTA = Ventral Tegmental Area.

**Fig. 5:**
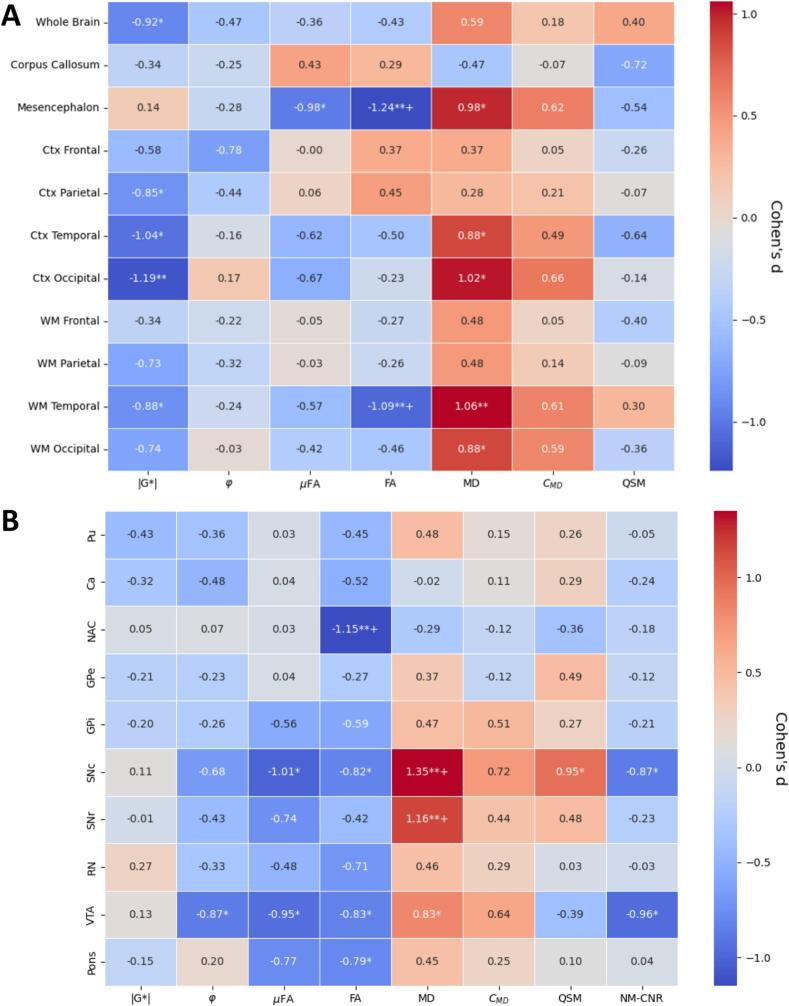
Standardized effect sizes (Cohen’s d) for the two groups (PD or HC). Median values in ROIs were adjusted for age effects by linear regression. (p values calculated by Mann-Whitney *U* test between groups with adjusted values, *=p < 0.05. **=p < 0.01. += p_FDR-corr < 0.05). A) shows major brain regions, and B) shows minor subcortical regions. Pu=Putamen, Ca=Caudate Nucleus, NAC=Nucleus Accumbens, GPe/i = Globus Pallidus external/internal, SNc/r=Substantia Nigra pars compacta/reticulata, RN = Red Nucleus, VTA = Ventral Tegmental Area.

The statistical analysis is based on median values within the regions of interest unless otherwise stated. Correlation strengths between the ROI values and age (see Fig. 2, Fig. 3, Fig. 4) were calculated using partial Pearson’s correlations where the values controlling for group effects (PD or HC) as covariate, and the associated p-values were calculated based on a two-sided *t*-test. Group difference effects (see Fig. 5) were calculated by first adjusting the ROI values for the linear effects of age using and then performed a Mann-Whitney *U* test on the residuals to calculate significance, and quantified the correlations using Cohen’s *d*. No statistical corrections were made due to gender, as there were relatively few women in the study (3 PD and 3HC). False discovery rates (FDR-BH) were also calculated per image modality to account for multiple comparison effects. These are shown in Fig. 4, Fig. 5, Fig. 7. Due to the exploratory nature of this study, and the low number of subjects, these corrections are, however, largely disregarded in the discussion. Instead, we focus on consistent and biologically meaningful trends relevant to the discussed effects.

## 3 Results

### 3.1 MR elastography

Fig. 2a shows the brain stiffness averaged for the HC and PD subjects after registering the individual maps to MNI space (Fonov et al., 2009). The stiffness for subjects with PD can be seen generally to be lower than for the HC. Fig. 2b shows the average stiffness and 2c shows average viscosity (averaged over the entire brain, excluding ventricles and CSF) as a function of the individual subject’s age for both HC (marked in black) and PD (marked in red); additional data for different subregions of the brain can be found in the Supplementary Information (SI) (Fig. SI1). There, it becomes apparent that the brain’s stiffness significantly decreases with age (p < 0.01; controlling for group), however, the effect is more prominent in PD subjects (p < 0.05; controlling for age). Viscosity also exhibits a decreasing trend with age (p < 0.01; controlling for group, however the difference between HC and PD is less obvious (p > 0.05; controlling for age).

### 3.2 MD-dMRI

Fig. 3a shows average μFA-maps for all HC and PD subjects, where some small local differences can be seen between the two groups. For example, there is slightly higher μFA in the frontal and temporal lobes for HC than for PD. Fig. 3b-e shows how the various MD-dMRI-derived parameters change, on average, across the whole brain as a function of age (see also Fig. SI1 for data in different subregions of the brain). It is clear that MD and, to a lesser degree, C_MD_ exhibits a positive correlation with age), whereas μFA exhibits a negative correlation with age (p < 0.01, p < 0.05 and p < 0.01 respectively; controlling for group). No significant correlation was detected for FA due to age. No significant differences can be seen between HC and PD for the whole brain averages of the MD-dMRI parameters.

### 3.3 Age effects

Fig. 4 shows correlations between age and the median values of MD-dMRI, MRE, QSM and NM-CNR of the subjects subdivided into various brain regions. For Fig. 4a, the brain regions were subdivided into the cortex (Ctx) and white matter (WM) of the four main lobes of the cerebrum (frontal, parietal, temporal, and occipital), and additionally, the corpus callosum and the mesencephalon. Fig. 4b shows correlations specifically for the subcortical structures. The correlations were calculated based on all subjects and corrected for group (PD or HC) as a covariate.

**Mechanical properties**

Apart from the corpus callosum and the mesencephalon, all structures soften with age (p < 0.01,p_FDR-corr < 0.05), with varying degrees, where the white matter regions of the cerebrum are the most affected. Fewer significant effects can be seen on the viscosity (φ), but still, the parietal lobe (p < 0.01,p_FDR-corr < 0.05), the WM temporal (p < 0.05,p_FDR-corr < 0.05), and the whole brain in general (p < 0.01,p_FDR-corr < 0.05) can be seen to significantly decrease in viscosity.

**MD-dMRI properties**

The μFA is decreased for all structures due to age (p < 0.01,p_FDR-corr < 0.05), with the WM of the frontal lobe most strongly affected. This effect is correlated with increased MD, which is significantly increased in all ROIs (p < 0.01,p_FDR-corr < 0.05),. The normalized variance of MD (C_MD_) is shown to most strongly correlate positively with age for the WM of the cerebrum and on the Ctx of the temporal lobe (p < 0.01,p_FDR-corr < 0.05), with little effect on the remaining structures. The macroscopic fractional anisotropy (FA) is mostly decreased in the parietal, frontal and temporal WM (p < 0.01,p_FDR-corr < 0.05), and to a lesser extent in the corpus callosum (p < 0.05,p_FDR-corr > 0.05), with little effect on other structures. Note that both μFA and FA are reduced for higher age for structures containing large WM tracts (cerebrum WM, corpus callosum, and mesencephalon), whereas in the Ctx, FA is not significantly dependent on age (FA values in the Ctx are very low in general, as expected).

**Subcortical effects**

Within the subcortical structures (see Fig. 4b), there is a significant softening (reduced |G*|, p < 0.05,p_FDR-corr > 0.05) of the Putamen (Pu), Caudate nucleus (Ca), Globus pallidus exterior (GPe), and Red Nucleus (RN). No significant effects were seen for the viscosity parameter. Some effects were found for the μFA on the Pu, and Ca (p < 0.05,p_FDR-corr > 0.05), but none for the FA. MD, however, displays several significant, strongly positive correlations with age within the Pu, Ca, NAC, RN and pons (p < 0.05 and p_FDR-corr < 0.05 for Pu and pons, p < 0.05 and p_FDR-corr < 0.05 for Ca, NAC, GPe, RN). C_MD_ only shows a significant decrease with age within the Ca (p < 0.01,p_FDR-corr < 0.05).

**Volume effects**

The tissue volume for several larger regions was compared for HC and PD patients, and although there was a general trend for smaller volumes in PD compared to HC, these differences were non-significant (two-sided *t*-test p > 0.05 for all comparisons).

### 3.4 Effects of Parkinson’s disease

Fig. 5 shows correlations (effect sizes, calculated as Cohen’s d between the two groups, and the associated p-values were calculated based on the Mann-Whitney *U* test) between the median imaging modality values in the different selected regions of the brain after correcting for age effects.

**Mechanical properties**

On average, over the whole brain, the only statistically significant change found was a decrease in stiffness (p < 0.05,p_FDR-corr > 0.05). Zooming in on the different regions, we found a softening of the temporal lobe (p < 0.05, p_FDR-corr > 0.05 for both WM and Ctx), the parietal Ctx (p < 0.05,p_FDR-corr > 0.05), and for the occipital Ctx (p < 0.01,p_FDR-corr > 0.05),

**MD-dMRI properties**

The softening of most of these regions (WM and Ctx for both temporal and occipital lobes) is also accompanied by an increase of MD (p < 0.01 for WM temporal, p < 0.05 for other structures with p_FDR-corr > 0.05). Interestingly, the mesencephalon, being strongly affected by PD, did not exhibit any significant softening in this study, as opposed to what was reported in previous studies (Lipp et al., 2018). The mesencephalon did however exhibit a significant reduction in µFA (p < 0.05,p_FDR-corr > 0.05) and FA (p < 0.01,p_FDR-corr < 0.05), and an increase of MD (p < 0.05,p_FDR-corr > 0.05).

**Subcortial effects**

For the subcortical regions (Fig. 5b), we see no significant softening due to PD, and little effect on viscosity in any region (except for the VTA, p < 0.05, p_FDR-corr > 0.05). There are, however, some significance for the μFA within the SNc and VTA (p < 0.05, p_FDR-corr > 0.05); some for FA within NAC (p < 0.01, p_FDR-corr < 0.05), the SNc and the VTA (p < 0.05, p_FDR-corr > 0.05). Strong positive correlations can be seen for group effects of MD within the SNc, SNr, and VTA (p < 0.01, p_FDR_corr < 0.05 for SNc and SNr; p < 0.05, p_FDR_corr > 0.05 for VTA). The C_MD_ follows the same pattern as the MD, but with very little to no significance.

**Symmetry effects**

An analysis of symmetrical effects across the brain was also conducted, comparing the right and the left hemisphere of the cerebrum for age and group effects (see Fig. SI2 and SI3 respectively). Few significant differences were detected except for the stiffness of the occipital lobe, where the left side exhibited much more softening due to PD than the right side.

### 3.5 QSM and NMI results

**Age effects**

As seen in Fig. 4a, levels of iron, as indicated by QSM levels in the brain, are increasing with age in some regions, most significantly in the parietal, frontal and occipital WM (p < 0.05, p_FDR_corr > 0.05), and decreasing with age in the corpus callosum (p < 0.05, p_FDR_corr > 0.05). Neuromelanin was excluded from the major brain region analysis since the FOV was limited to a slab crossing the midbrain, and thus most global structures, such as the occipital lobe, are only partially included and may vary between subjects. Within the subcortical structures (Fig. 4b), QSM only shows a significant negative correlation with age within the Ca (p < 0.01, p_FDR_corr > 0.05), whereas NM-CNR shows significant negative correlations for the NAC (p < 0.01, p_FDR_corr > 0.05).

**Group effects**

For group differences between PD and HC (see Fig. 5a), there are no significant differences on the global level for QSM, although the QSM signal in the corpus callosum and Ctx temporal exhibits large effect sizes (|Cohen’s d|>0.6). Zooming in on the subcortical regions (Fig. 5b), however, we detect a significant increase of QSM in the SNc and a decrease of NM-CNR in the SNc, and the VTA (p < 0.05, p_FDR_corr > 0.05 for all).

### 3.6 Correlations between MRE, MD-dMRI and QSM

**Fig. 6:**
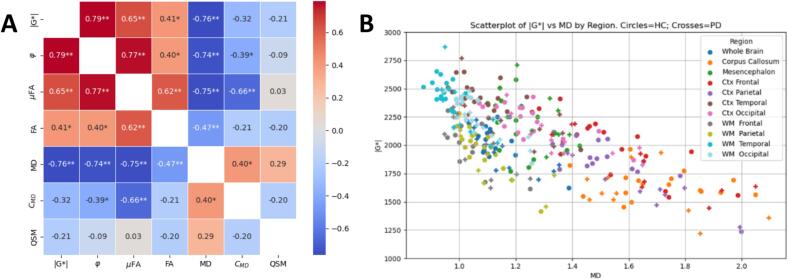
A) Correlations between imaging modalities for all subjects’ whole brain average. Values in each box show the Pearson correlation coefficient after correction for group (HC vs. PD) effects. *=p < 0.05. **=p < 0.01. += p_FDR-corr < 0.05. B) Stiffness as a function of MD for all subjects divided into different regions, as listed in the legend. Circular marks signify HC, plus-signs signify PD subjects.

Correlations between all image modalities were calculated for the whole brain to assess the relationship between MRE, MD-dMRI and QSM parameters. Fig. 6a shows these correlations for the whole brain averages. Fig. 6b shows an example correlation plot for several regions overlaid, specifically for |G*| as a function of MD. There exists a clear negative correlation between MD and both stiffness and viscosity and a clear positive correlation between μFA and both stiffness and viscosity. These correlations are well in line with the previous results (Le Bihan et al., 2017), who showed a significant negative correlation between the apparent diffusion constant and the stiffness of the liver. Notice also that the correlation between C_MD_ and stiffness is weak, and that QSM has little correlation to any of the other parameters.

### 3.7 Correlations with clinical scores (UPDRS & MoCA)

**Fig. 7:**
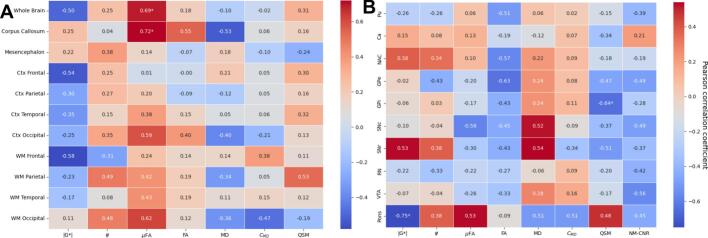
Correlations (Pearson coefficients) between changes in the measured values with total UPDRS score (age-corrected) for the PD cohort (excluding HC). A: global structures. B: subcortical structures. *=p < 0.05. **=p < 0.01. += p_FDR-corr < 0.05, Pu=Putamen, Ca=Caudate Nucleus, NAC=Nucleus Accumbens, GPe/i = Globus Pallidus external/internal, SNc/r=Substantia Nigra pars compacta/reticulata, RN = Red Nucleus, VTA = Ventral Tegmental Area.

The various extracted measurements were also evaluated against the different clinical scores (UPDRS 1–4 and MoCA) of the PD patients. Pearson correlations between the clinical scores and imaging modality parameters were calculated after correcting for age and are shown in the Supplementary Information (Figs. SI4-SI8). Fig. 7 exemplifies these correlations by showing the Pearson correlations between the various imaging modalities and the total UPDRS score (sum of UPDRS 1–4), where most of the correlations are relatively insignificant for the global regions (see Fig. 7a), showing only a significant increase in μFA for the whole brain and corpus callosum. Zooming in to the subcortical regions (see Fig. 7b), we find negative correlation between total UPDRS and |G*| within the pons, and negative correlation between total UPDRS and QSM within the GPi (p < 0.05,p_FDR_corr > 0.05 for all mentioned correlations). A few relevant highlighted results can be seen for the individual clinical scores (Figs. SI4-SI8), such as a significant softening within the frontal lobe (p < 0.05 for Ctx and p < 0.01 for WM) with increased UPDRS-1, and an overall increasing trend of MD with decreasing MoCA scores, however given the low number of data-points for these correlations the statistical power is very low.

## 4 Discussion

### 4.1 MRE results

The reduction of shear stiffness with age has previously been shown and, for example, Sack and co-workers (Sack et al., 2009) found that |G*| is reduced with 15 Pa per year for healthy subjects. This trend is consistent with the present results (see Fig. 2B). In PD patients, |G*| declines at an even faster rate by approximately 20 Pa per year (Fig. 2B), suggesting an accelerated aging effect due to the disease.

Only a few studies have investigated the effects of PD on the mechanical properties of the brain using MRE in humans. In particular, Lipp et al (Lipp et al., 2013, 2018) evaluated this relationship in two cohorts of 18 PD vs 16 HC (Lipp et al., 2013) and 17 PD vs 12 HC (Lipp et al., 2018), respectively.^2^ Notice that those two studies were carried out with different scanner settings, which may have a significant effect on the measured viscoelastic properties(Hiscox et al., 2016). For example, they only acquired a part of the brain (6 cm slab through the central cerebrum in the 2013 study and a 2 cm slab in the 2018 study), and they used a different frequency (50 Hz) in the 2013 paper and multifrequency acquisition (6 frequencies at 30–60 Hz) in the 2018 paper, which makes it difficult to make exact quantitative comparisons.

Another relevant study on PD using MRE is that of Hain and co-workers (Hain et al., 2016), who rather selectively killed dopaminergic neurons in the substantia nigra of mice (by injection of 1-methyl-4-phenyl-1,2,3,6-tetrahydropyridin hydrochloride (MPTP)) and measured the effect of the resulting viscoelastic parameters. They found that the effects of MPTP induced neurodegeneration of dopaminergic neurons in the SN, which correlated with decreased viscosity and decreased elasticity to a less significant extent.

In the present study, we observed that the viscosity (φ) decrease with increasing age − particularly in the white matter- indicating that these regions become more elastic-dominant (i.e. relatively less viscous) as they soften. At the whole brain level, |G*| and φ were positively correlated (see Fig. 6); a correlation that is seen particularly for the cerebral WM (p < 0.01) and in the corpus callosum (p < 0.05). In contrast, no significant correlation was seen within the mesencephalon or cerebral cortex (p > 0.05, data not shown). It is important to note that φ is typically more sensitive to noise (Dittmann et al., 2016), particularly in regions with spatial inhomogeneity or near tissue boundaries. For example, the cortical surface is prone to partial volume effects and inversion artifacts due to local violations of the homogeneity assumption. Thus, the correlations between φ and age (or group effects) seen in these regions should be interpreted cautiously.

Lipp et al propose in their 2013 study (Lipp et al., 2013) that reduced stiffness with unchanged viscosity implies that the mechanical scaffold of the tissue is weakened while its architecture remains intact. While we observed a non-significant trend showing reduced φ in PD, the noise levels made this result unreliable.

As mentioned, our data show a general reduction in brain stiffness in PD, in agreement with the findings of (Lipp et al., 2013, 2018) (comparisons between the present study with these two studies by Lipp et al. can be found in the supplementary information) and analogous to the findings in a mice model by (Hain et al., 2016). Although the absolute stiffness values from (Lipp et al., 2018) deviated from our results (and their previous study (Lipp et al., 2013)), the relative changes due to PD are comparable. For example, Table SI2 shows that in the frontal region, we detect an 8 % reduction (p = 0.04) for the HC/PD group effect (using an ANOVA test with age as a covariate) compared to (Lipp et al., 2018), who reported a 14 % decrease in |G*| (p = 0.02) in the frontal lobe (although they used a different segmentation method). (Lipp et al., 2018) also detected a significant decrease of |G*| in the mesencephalon, whereas we detected no significant changes of the mesencephalon in the present study. They found no significant effects due to PD on φ (except for the striatum), which mostly agrees with the present findings (see Fig. 5). The comparison of our results and those of (Lipp et al., 2013) reveals several discrepancies, showing consistently stiffer measurements in the present study, which have many potential explanations. Differences in cohorts is one possible explanation since Parkinson’s is a disease with heterogeneous effects on the brain. However, both Lipp et al’s cohorts have similar age, disease duration, and UPDRS-III scores as the present study, which makes methodological effects a more plausible explanation. Differences in delineation in small structures, such as the mesencephalon, may impact measurements due to partial volume effects. Differences in vibration frequency can also explain a large part of the quantitative differences as lower frequencies leads to lower stiffness values (see e.g. (Dittmann et al., 2016)).

### 4.2 MD-dMRI results

Conventionally, FA and MD (which is qualitatively similar to apparent diffusivity coefficient (ADC)) have been extensively used to study all kinds of neuropathologies, including PD (Atkinson-Clement et al., 2017, Basser, 1995, Cochrane and Ebmeier, 2013). These studies have provided insights into how white matter tracts are affected by the disease. However, MD and FA are average measures of the entire voxel and yield limited information about the microscopic nature of the investigated tissue. Thus, several studies have also included more advanced dMRI techniques, such as multi-shell dMRI and MD-dMRI to study PD (see e.g., (Kamagata et al., 2014, Kamiya et al., 2020, Surova et al., 2016, 2018)) motivated by the fact that measures like μFA (or mean kurtosis-measurements) are more sensitive to microscopic changes than conventional measures like FA and MD (Kamagata et al., 2014, Surova et al., 2018).

Fig. 4 shows how the microstructural properties overall are altered due to aging. A clear reduction of the μFA and an increase of the MD in most structures were found. Furthermore, we report a decrease of the FA for most WM structures, the CC, and an increase of the C_MD_ of the WM of the frontal, temporal, occipital and parietal lobes plus in the Ctx of the temporal lobe. The fact that μFA decreases in the Ctx but not FA can be seen because of the overall low values of FA within the cortex. These results are generally well in line with previous the results (Benitez et al., 2018, Kamiya et al., 2020, Lawrenz et al., 2016), who studied the WM of the above-mentioned structures and indeed also detected the same trend, i.e., decreased FA and μFA, and increased MD and C_MD_.

For changes due to PD, (Kamiya et al., 2020) and (Surova et al., 2016) also studied the effects of a PD diagnosis on microstructural properties. Kamiya et al.(Kamiya et al., 2020) showed significant effects of the MD in the WM of the parietal, frontal, and occipital lobe, whereas we show a similar level of significance only for the temporal and occipital lobes (WM and Ctx) and the mesencephalon. (Surova et al., 2016) showed a significant effect with increasing MD for the superior longitudinal fasciculus, a WM tract that connects the temporal, occipital, parietal and frontal lobe, which also qualitatively agrees with the present results.

(Kamiya et al., 2020) showed very little effect of the μFA (no significant differences between HC and PD in their investigated regions), which is largely in line with what is reported here (although some significant effect was found in the mesencephalon). In the present study, a significant decrease of µFA and FA between HC and PD was found in the mesencephalon and in the temporal WM for the FA signal. This is partly in line with (Surova et al., 2016), who only found a significant decreasing effect on the putamen and the thalamus, and (Kamiya et al., 2020) reported some effect on the anterior limb of the internal capsule.

### 4.3 QSM and NM-MRI results

Several previous studies have investigated the effects of PD on QSM where it is consistently found that QSM levels increase within the substantia nigra (see e.g., (Acosta-Cabronero et al., 2017, Andersson Forsman et al., 2024, Sjöström et al., 2017, Thomas et al., 2020)), consistent with the present findings. (Acosta-Cabronero et al., 2017) also detected a significant increase of QSM due to PD (after correcting for age effects) within the cortical areas of the lateral occipital, posterior parietal, rostral middle prefrontal, middle temporal gyrus and the hippocampus, which were not apparent in the present study. NMI showed a significant decrease in neuromelanin within the SNc, which is consistent with previous studies (Biondetti et al., 2021, Matsuura et al., 2016, Takahashi et al., 2018). Changes in both QSM and NMI within the mesencephalon thus agree with what is expected from the literature, which is encouraging for the further use of QSM and NMI as diagnostic markers for PD. It is worth noting that although the mesencephalon in general, and the SN in particular, is usually found to be significantly affected by PD, there were few signs of changes due to mechanical properties in these regions due to PD (see Fig. 5b), except a decrease in viscosity due to PD within the VTA. On the other hand, MD-dMRI properties were generally strongly affected, most likely due to neural atrophy (decrease of μFA and FA, increase of MD and C_MD_). In fact, these modalities were shown to be more significantly affected than QSM and NMI for several regions within the present study.

### 4.4 Microstructural mechanisms of aging and PD

By combining the reported MRE and MD-dMRI-derived parameters, one can better understand the biological mechanisms behind the changes in brain tissue. If we, for example, analyze the WM-regions (which shows very clear signs of softening both due to age and PD effects), we can see that the tissue in these regions decreases in both μFA and FA and increases in MD and C_MD_ due to age. This combination is consistent with neuronal loss due to age: fewer fibers generally results in a lower μFA and a lower FA (assuming we compare to a scenario with generally aligned fibers), which is replaced by extracellular free water, resulting in higher MD and C_MD_ (if the replacement/atrophy occurs heterogeneously). In contrast, these regions appear to be less significantly affected for μFA, FA and C_MD_ when observing effects due to PD. This implies that the neuronal fibers are more intact, or at least that the decrease is too subtle to be significant, whereas the increase of MD implies more free water in the system. This could be explained by, for example, a decrease in the number of non-directional cells (e.g., glial cells) surrounding the axons, which would have a small effect on FA and µFA while allowing more extracellular water in the volume. The cortical gray matter is affected by age- and group-differences in a similar manner to the WM, with the exception that FA remains unchanged for aging effects. This may simply be due to the overall small FA in the cortex, as there is little global anisotropy there, but it should also be noted that there is more noise associated with these regions due to partial volume effects. In the corpus callosum, we note a clear effect of decreasing FA and μFA in combination with increased MD (for aging effects), while little effect is seen on its stiffness. If this motif from the MD-dMRI parameters implies neuronal atrophy or axonal degradation, one would, in contradiction to observed measurements, also presume a loss of stiffness in this region. This discrepancy could possibly be attributed to some form of axonal degradation that does not significantly alter the mechanical properties, such as demyelination, although further studies are required to make any claims to the underlying reason for this effect. Similarly, the mesencephalon exhibits a decrease of µFA and increase of MD due to age, not associated with a change in mechanical properties. These alterations can also be seen for group comparisons, however with the addition of a significant reduction of FA for PD patients, again implying that there is significant axonal degradation (loss of fiber tracts) without any major alterations to the mechanical properties.

Multiple studies have previously shown different effects on the white matter and the cortical grey matter due to PD that fits with the reported findings: Pieperhoff et al (Pieperhoff et al., 2022), for example, showed that the volume of the temporal and occipital lobe, in particular, is reduced over time for PD patients, indicating that these structures are indeed particularly affected by the disease.^3^ Previous studies using dMRI (Kamiya et al., 2020, Surova et al., 2016) have also shown some microstructural alterations in the brain due to PD. However, these have been relatively small effects, which fit largely with the presented results. The presented results in this study gives an analysis of how the microstructural mechanisms correlate with age and PD, and how these correlations are associated with the mechanical properties of the tissue. However, further data and analysis are required to support any direct evidence for the mechanisms discussed.

## 5 Limitations

There are several limitations to point out regarding the presented work. One major limitation of this study is the relatively low number of subjects, which may give rise to statistical uncertainties. This is particularly true for all UPDRS correlations, where we were limited to the PD cohort, consisting of 12 subjects. It should also be stated that UPDRS was performed in ON state and results are a combination of PD *per se* and medication. Another limitation is the relatively low resolution of MRE (3 mm) and MD-dMRI (2.5 mm), which may give rise to partial volume effects; this is a limitation that will have an effect on the whole brain, where the properties of varying regions spill into each other, but most significantly is this a problem for regions close to CSF where the shear stiffness, for example, is not applicable. Thus, some spurious results may affect the estimations at the cortical surface, for example. This is also true for the analysis of small subcortical structures (e.g., the SNc). The accuracy of the viscoelastic parameters is also limited by the inversion method that relies on common assumptions of the material, such as isotropic viscoelastic properties and local homogeneity (Murphy et al., 2013, Oliphant et al., 2001). These assumptions are imposed by solving the Helmholtz equation regionally within a kernel of a fixed size and the method is thus sensitive to errors in regions where these assumptions break; for example, near tissue boundaries, such as the cortical surface. This method is also inherently more sensitive to noise than many other common methods as it depends on second or third order derivatives of the displacement field (Murphy et al., 2019). It is, however, a commonly used method, implemented in clinical scanners, which is robust, fast and useful for comparisons to similar studies. In future work, errors associated with this method could be mitigated using more advanced inversion methods, such as finite element-based non-linear inversion techniques (McGarry et al., 2012) which show improved accuracy in heterogeneous regions, or novel techniques such as travelling wave expansion (TWE) (Ma et al., 2025) or using neural networks, as implemented in for example the works of Scott et al. (2020), Bustin et al. (2025) or Ma et al. (2023), which improves stiffness estimation accuracy without vast computational resources.

Another major limitation of this study is that although we suggest plausible mechanisms coupling mechanical properties of the brain to structural changes (e.g. neuronal loss), these interpretations are yet to be validated. Future work related to this study is to further develop computational models that more accurately link the microstructure of the brain to its mechanical properties and validate these couplings through experiments (e.g. via *ex vivo* histology measurements).

## 6 Summary and conclusions

This study presents an analysis of how PD affects the whole brain using a combination of different modalities, namely MRE, MD-dMRI, QSM, and NMI. Although multiple studies have investigated neurodegenerative disorders in general using these modalities separately, this is, to our knowledge, the first study that investigates how these combined modalities change due to Parkinsons disease. The main findings are:

1. The regions that are most significantly softened by PD are the temporal and the occipital lobes.
2. These changes are associated with an increase in the MD, where most other microstructural properties remain insignificantly altered (except a decrease of FA in the WM temporal). This suggests that neural atrophy might not be the predominant mechanism behind the decrease in stiffness.
3. The mesencephalon (and most of its substructures) exhibits alterations of the microstructural properties which is more consistent with neural atrophy. However, these changes are not generally accompanied by changes in the mechanical properties.
4. Multiple regions of the brain are softened due to age, and we show that these changes possibly correlate mainly with a microstructural change that can be attributed to neural atrophy.
5. Mechanical properties are strongly correlated with μFA and MD.

## CRediT authorship contribution statement

**Christoffer Olsson:** Writing – review & editing, Writing – original draft, Visualization, Software, Methodology, Investigation, Formal analysis, Conceptualization. **Mikael Skorpil:** Writing – review & editing, Methodology, Conceptualization. **Per Svenningsson:** Writing – review & editing, Methodology, Funding acquisition, Conceptualization. **Rodrigo Moreno:** Writing – review & editing, Supervision, Resources, Project administration, Methodology, Funding acquisition, Conceptualization.

## Declaration of Competing Interest

The authors declare that they have no known competing financial interests or personal relationships that could have appeared to influence the work reported in this paper.

## Data Availability

The MR imaging data in this study are subject to ethical and legal restrictions and cannot be shared publicly, however anonymized data can be made available upon reasonable request to researchers who meet the legal requirements. Please contact the corresponding author for more information.

## Footnotes

1 The FOV was occasionally, for some subjects, cut off at the inferior part of the cerebellum, which is why this part of the anatomy is omitted from any analysis.

2 Both studies included a cohort of patients with progressive supranuclear palsy (PSP), which are excluded from any comparisons presented here. PD patients in both Lipp’s studies had comparable age-span (63:67.3), disease duration (9.3:9.8), and UPDRS-3 scores (16.7:32.2); (2013 study: 2018 study).

3 They also showed an effect in the inferior parietal lobule, the insula, putamen and the nucleus basalis of Meynert.
